# Preoperative smoking cessation counseling activities of anesthesiologists: a cross-sectional study

**DOI:** 10.1186/s12871-015-0036-6

**Published:** 2015-04-28

**Authors:** Matilde Zaballos, Maria Iluminada Canal, Rocío Martínez, Maria José Membrillo, Francisco J Gonzalez, Hugo D Orozco, Francisco J Sanz, Maite Lopez-Gil

**Affiliations:** 1 Department of Toxicology, Faculty of Medicine Complutense University, Madrid, Spain; 2 Department of Anaesthesiology, Hospital Universitario Gregorio Marañón, Madrid, Spain; 3 Head of the Department of Anesthesiology, Hospital Universitario Gregorio Marañón, Madrid, Spain

**Keywords:** Smoking, Anesthesia, Preoperative smoking interventions

## Abstract

**Background:**

Smokers undergoing surgery are at a higher risk of complications than non-smokers. Preoperative evaluation by an anesthesiologist could provide an excellent opportunity to promote smoking cessation. Previous surveys of anesthesiologists have found that self-reported smoking cessation counseling rates have room for improvement, but no study has surveyed patients to obtain more accurate estimates.

**Methods:**

A single-center study was conducted from January 2010 to June 2010 in a tertiary teaching hospital. A telephone survey was conducted, which included all adult cigarette smokers who visited the preoperative anesthesia clinic. The survey recorded anesthesiologist-delivered interventions to help patients quit smoking before surgery. At the end of the study period, the self-reported smoking cessation counseling of the anesthesiologist was evaluated by questionnaire.

**Results:**

One thousand one hundred and sixty-five patients were evaluated, of which 217 were current smokers with a median pack-year of 15 (interquartile range 5.25–30.00) and 34% were scheduled to undergo major surgery. With regard to preoperative interventions, most anesthesiologists (85%) asked about smoking status, although only 31% advised patients about the health risks of smoking and 23% advised patients to quit before surgery. Provision of assistance to help patients quit was provided in 3% of cases. By contrast, 75% of anesthesiologists stated that they frequently or almost always advised patients about the health risks of smoking.

**Conclusions:**

This study shows significant discrepancies between direct patient surveys of preoperative smoking cessation counseling activities by anesthesiologists and the self-reported perceptions of the anesthesiologists. Future studies are urgently needed to evaluate the provision of educational materials and other interventions to improve smoking cessation counseling rates among anesthesiologists and to narrow these discrepancies.

## Background

Cigarette smokers are at an increased risk of severe postoperative pulmonary and cardiovascular complications and impaired healing of bones and surgical wounds [1-4]. However, smoking is often seen as a risk factor that is not easily altered, although several studies suggest otherwise [5]. Some postoperative adverse effects of smoking are diminished following cessation before surgery, and the longer the duration of preoperative abstinence, the better, especially with regard to pulmonary complications [1,6,7].

Surgery provides an excellent opportunity to motivate patients to quit smoking; however, previous studies have shown that anesthesiologists might not fully appreciate the risk of smoking in the perioperative period [8-11]. A recent pilot study evaluated the feasibility and acceptability of tobacco cessation interventions in anesthesiology practice, following an educational program based on a variety of materials in 14 American anesthesiology practices [12]. Three months after implementation, a survey showed that the “Ask-Advise-Refer” approach (Specifically, ask the patient about his/her smoking history, advise the patient that continuing to smoke increases the risks of surgery, and refer the patient for smoking cessation counseling) was adopted frequently: 91% asked, 79% advised and 58% referred [12]. However, although this study was large, it focused only on anesthesiologist self reports on tobacco counseling, and therefore it did not provide information about the actual patient’s perspective of counseling behaviors.

Counseling of smokers as part of the anesthesiologist’s preoperative evaluation has never been evaluated by a direct patient survey. As an initial step to promoting tobacco control interventions in our surgical patients, we investigated interventions by anesthesiologists in adult patients who smoked and were scheduled for elective surgery. As a secondary outcome, we determined the attitudes of the same anesthesiologists using a survey modified from a questionnaire designed to study this issue in other countries [9-11].

We hypothesized that the actual tobacco counseling delivered by anesthesiologists as reported by patients would be lower than that reported in previous surveys, and inferior to that self-reported by the anesthesiologists assessed in the present report.

## Methods

Between January 10, 2010 and June 30, 2010, a single-center, cross-sectional telephone survey of patients was performed to study the tobacco cessation interventions in anesthesiology practice in a tertiary teaching hospital.

The study was approved by the Institutional Review Board of Hospital Gregorio Marañón (Madrid, Spain) in November 2009. Patients included were required to be able to provide informed verbal consent. The staff anesthesiologists consented to participate in the study but they were blind to its objectives.

All patients undergoing a preoperative medical examination in the anesthesia clinic unit were eligible for enrolment. In our hospital, the pre-anesthetic evaluation data are automatically recorded in a specially designed online form, which enables the data to be reviewed after the preoperative visit. Patients were excluded if they had poor language comprehension, a mental illness or had experienced language difficulties. We also excluded duplicate encounters.

Residents and medical students (fifth and sixth years of medical school) conducted a telephone survey of all adult cigarette smokers who visited the preoperative anesthesia clinic. They received extensive training on the research study.

The information was gathered on different days during the study period to ensure that the practice of a wide sample of anesthesiologists was observed. According to similar studies, a minimum of 15 smokers evaluated by each anesthesiologist were required [13,14]. We excluded the cases of pediatric and obstetric anesthesiologists.

The telephone survey was conducted over a period not exceeding 24 hours after the preoperative visit, and we allowed three telephone calls if no response to the first attempt was attained. The investigators collected information relevant to the study, including baseline characteristics and whether the patient was a current smoker, ex-smoker or non-smoker. Smoking status was classified as follows: current smoker (having smoked within 1 month of the preoperative evaluation), ex-smoker (not having smoked within 1 month of the preoperative evaluation) and non-smoker (never having smoked cigarettes). Information obtained via telephone interview included questions asked to confirm smoking habits. In addition, smokers were asked whether during the pre-anesthetic visit, the anesthesiologist had discussed the adverse effects of tobacco smoking in the perioperative period. After recruitment, when all the patients had been included according to the statistical sample size calculation, we interviewed the anesthesiologists about their smoking cessation practices in the pre-anesthetic visit.

### Assessment of preoperative factors

We recorded age, sex, height, weight, American Society of Anesthesiologists (ASA) classification of physical status, comorbidities and scheduled surgical procedure.

### The anesthesiologist’s assessment of the patient’s smoking behavior

The interviewer asked the patient the following questions concerning whether smoking had been discussed during the pre-anesthesia visit:

- Did the anesthesiologist advise you about the health risks of smoking and perioperative complications (i.e. cardiovascular, respiratory, and wound and bone healing)?
- Did the anesthesiologist advise you about the benefit of preoperative abstinence (i.e. changes in the cardiovascular system, such as a decrease in heart rate and blood pressure, improved lung function and a reduced risk of wound complications)?
- Did the anesthesiologist provide you with counseling or other resources such as medications, prescriptions for medications, educational material or a referral for nicotine dependence treatment?

### Assessment of perioperative prescriptions

The pre-anesthetic online record was used to review whether the anesthesiologist had prescribed β_2_ adrenergic aerosols and corticosteroids as premedication before the induction of anesthesia. This prescription is the current practice in our department for patients with bronchial hyperactivity, including smokers, to prevent bronchospasm associated with airway manipulation.

Last, we evaluated whether the anesthesiologist’s assessment varied according to his/her own smoking status, the patient’s ASA status, the type of surgery (major vs. minor), the age and body mass index of the patient, or the need for tracheal intubation.

### Statistical analysis

Statistical analysis was conducted using IBM SPSS, version 20.0, Spain. Categorical variables are summarized as frequencies and percentages, and continuous variables as means and standard deviations. Descriptive analyses of the preoperative assessment were prepared and represent the primary focus of this study; each item in the preoperative evaluation was coded into discrete categories (yes/no).

The chi-squared and Fisher exact test were used to evaluate whether anesthesiologists’ attitudes and recommendations differed according to the patients’ ASA status, the need for tracheal intubation, and whether surgery was major (e.g., intraperitoneal and intrathoracic procedures, cardiac bypass surgery, suprainguinal vascular procedures, orthopedic surgery, head and neck surgery and prostate surgery) or minor (e.g., endoscopic and superficial procedures, cataract surgery, breast surgery and ambulatory surgery).

Questions about the anesthesiologists’ current practice were classified using four options (never, sometimes, frequently, and almost always) and are presented as frequencies and percentages. P values <0.05 were considered significant.

Previous studies have reported that the proportion of anesthesiologists that always advise quitting smoking varies between 57% and 79% [9-11]. Accepting an alpha risk of 0.05 and a beta risk of 0.2 in a two-sided test, 191 participants were necessary in the observed group to recognize a difference ≥10%. A mean advising rate of 60% in the reference group has been estimated. A sample size of 217 was chosen to allow for potential drop-outs. In addition, we required a minimum of 15 smoking patients evaluated by each anesthesiologist.

## Results

**Figure 1:**
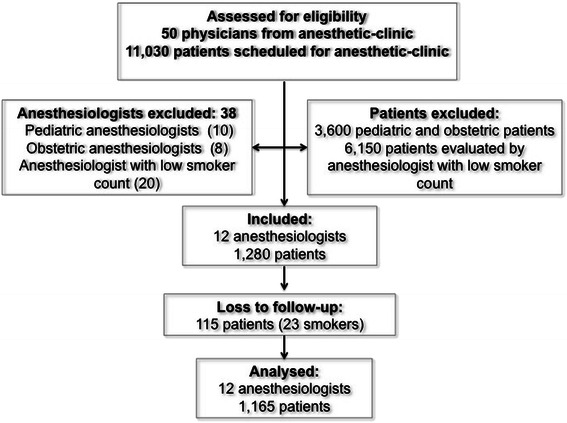
Flow diagram of the progress anesthesiologists and patients through the study.

From January to June 2010, we assessed 1165 preoperative evaluations in which 217 current smokers were identified (18.6%). To have sufficient smoking patients evaluated by each anesthesiologist (a minimum of 15 patients), we included the consecutive patients evaluated by 12 anesthesiologists from a total of 50 (Figure 1). These patients were predominantly male (58%) and middle aged (Table 1). The mean smoking time in current smokers was 24.5 ± 8.6 years (95% confidence interval 22.6 to 26.47) and the median number of pack-years was 15 (interquartile range 5.25–30.00). The demographic data of the anesthesiologists are presented in Table 2: 16% were current smokers, 34% were ex-smokers and 50% were non-smokers.

**Table 1:** **Patient baseline characteristics**

	Patients (n=217)
Age, years	49±15
Gender, n (%)	
Male/Female	125 (58%)/92 (42%)
Body mass index, kg/m^2^	25.16±4.72
ASA status, n (%)	
I	81 (37)
II	94 (43)
III	40 (19)
IV	2 (1)
Medical history	
COPD	40 (18%)
Cardiac disease	23 (11%)
Hypertension	44 (20%)
Diabetes mellitus	15 (7%)
Pack-years*	
Mild: 0-20	131 (65,5%)
Moderate: 21-40	52 (26%)
Heavy >40	17 (8%)

Data are given as mean ± SD, or number (percentage). Cardiac disease includes coronary artery disease, congestive cardiac failure and arrhythmias.

COPD: chronic obstructive pulmonary disease. *Cigarettes per day/20 × years of smoking. In 17 patients, the number of years of smoking is unknown.

**Table 2:** **Demographic data of anesthesiologists**

Characteristics	N=12 (%)
Age, y	
Under 35	-
35-44	4 (33)
44-54	8 (67)
≥55	-
Gender, n (%)	
Male	9 (50)
Female	3 (50)
Years in practice	
≤5	-
6-10	5 (42)
11-20	5 (42)
≥21	2 (16)
Current smoking status	
Smoker	2 (16)
Ex-smoker	4 (34)
Nonsmoker	6 (50)

### Patient reports of the advice received from anesthesiologists

**Figure 2:**
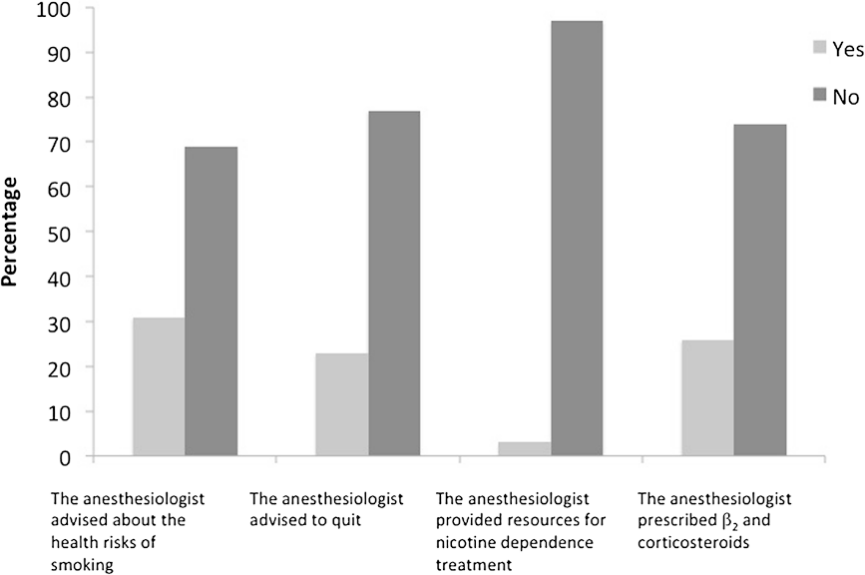
Summary of the anesthesiologists’ preoperative counseling obtained from patients’ telephonic interview.

Figure 2 summarizes the preoperative evaluation by the anesthesiologists according to the patient survey findings. In the 217 current smokers, anesthesiologists advised on the health risks of smoking in 31% and recommended quitting tobacco smoking before surgery in 23%. However, few anesthesiologists provided counseling or other resources to help their patients quit (only 3% of cases). The self-reported questions answered by anesthesiologists are shown in Table 3. There was a large difference between the counseling recorded as being given by anesthesiologists and that actually reported as being received by patients (from 0% to 53% in the ones that advised on the health risks of smoking and from 6% to 44% in those who recommended quitting tobacco smoking before surgery). Table 4 shows the data self-reported by anesthesiologists and the corresponding responses reported by their patients. We were unable to identify any patient or physician characteristics that influenced whether a patient received counseling (Table 5).

**Table 3:** **Summary of the self-reported questionnaire administered to the 12 anesthesiologists evaluated in the study**

	Never	Sometimes (<25% of the time)	Frequently (25-75% of the time)	Almost always (>75% of the time)
Do you ask your patients if they smoke cigarettes, cigars, or a pipe?	0	0	8	92
Do you advise your patients about the health risks of smoking?	0	25	42	33
Do you advise smokers to quit?	0	25	50	25
Do you provide resources to smokers to help them to quit?	25	75	-	-

**Table 4:** **Comparison of the responses to different questions reported by anesthesiologists and their patients**

	How often do you:	Self-reported by anesthesiologist	Reported by patients
Anesthesiologist 1	Advise your patients about the health risks of smoking?	Frequently	6%
	Advise smokers to quit?	Frequently	6%
	Provide resources to help them quit?	Sometimes	0%
Anesthesiologist 2	Advise your patients about the health risks of smoking?	Frequently	50%
	Advise smokers to quit?	Almost always	44%
	Provide resources to help them quit?	Sometimes	6%
Anesthesiologist 3	Advise your patients about the health risks of smoking?	Sometimes	30%
	Advise smokers to quit?	Sometimes	25%
	Provide resources to help them quit?	Sometimes	15%
Anesthesiologist 4	Advise your patients about the health risks of smoking?	Frequently	33%
	Advise smokers to quit?	Frequently	20%
	Provide resources to help them quit?	Sometimes	7%
Anesthesiologist 5	Advise your patients about the health risks of smoking?	Sometimes	48%
	Advise smokers to quit?	Frequently	22%
	Provide resources to help them quit?	Sometimes	4%
Anesthesiologist 6	Advise your patients about the health risks of smoking?	Almost always	27%
	Advise smokers to quit?	Almost always	7%
	Provide resources to help them quit?	Never	0%
Anesthesiologist 7	Advise your patients about the health risks of smoking?	Frequently	46%
	Advise smokers to quit?	Sometimes	44%
	Provide resources to help them quit?	Never	0%
Anesthesiologist 8	Advise your patients about the health risks of smoking?	Frequently	53%
	Advise smokers to quit?	Frequently	27%
	Provide resources to help them quit?	Sometimes	0%
Anesthesiologist 9	Advise your patients about the health risks of smoking?	Sometimes	44%
	Advise smokers to quit?	Sometimes	39%
	Provide resources to help them quit?	Sometimes	6%
Anesthesiologist 10	Advise your patients about the health risks of smoking?	Almost always	28%
	Advise smokers to quit?	Almost always	11%
	Provide resources to help them quit?	Sometimes	0%
Anesthesiologist 11	Advise your patients about the health risks of smoking?	Almost always	11%
	Advise smokers to quit?	Frequently	6%
	Provide resources to help them quit?	Never	0%
Anesthesiologist 12	Advise your patients about the health risks of smoking?	Almost always	0%
	Advise smokers to quit?	Frequently	16%
	Provide resources to help them quit?	Sometime	0%

**Table 5:** **Summary of the anesthesiologists’ counseling to smokers according to the nature of the surgery scheduled**

	Major surgery (n=74)	Minor surgery (n=143)	P
The anesthesiologist advised the patient about the health risks of smoking (%)	27	34	0.32
The anesthesiologist advised smokers to quit (%)	26	21	0.43
The anesthesiologist provided resources such as referral for nicotine dependence treatment (%)	4	3	0.61
The anesthesiologist prescribed β_2_ adrenergic aerosols and corticosteroids as premedication before induction of anesthesia (%)	32	23	0.13

Obtained from the patient survey and the data in the online form.

## Discussion

To our knowledge, this study is the first to analyze the extent of preoperative smoking cessation counseling activities by anesthesiologists as reported by their patients. The results of our survey are disappointing, showing that very few smokers receive appropriate smoking cessation counseling from anesthesiologists before surgery. This observation was not affected by type of surgery, the patient’s smoking status, ASA classification, or the need for intubation.

Clinicians in diverse specialties can play an important role in helping patients quit smoking. Although surgeons and anesthesiologists can encourage patients awaiting surgery to quit smoking before their operation, our research shows that this opportunity is not taken advantage of in clinical practice.

Previous reports have analyzed the practice of anesthesiologists in terms of preoperative smoking cessation counseling; however, a direct telephone survey of adult cigarette smokers who have been evaluated in the preoperative anesthesia unit has never been performed. An anonymous survey performed by Warner et al. in 2004 among 328 active anesthesiologists and 299 general surgeons showed that clinicians in the USA were usually more attentive than Spanish clinicians to smokers. [9] This difference is particularly evident when we compare the proportion of anesthesiologists who frequently or almost always advised patients about the health risks of smoking in the study by Warner et al. and our study: 52% vs. 31%, respectively. In addition, the proportion of anesthesiologists who frequently or almost always advised patients to quit was 57% vs. 23% in our study. As for the percentage of anesthesiologists who provided resources to help smokers quit, the result was discouraging both in Warner’s research and ours: 5% and 3% of anesthesiologists frequently or almost always provided resources to their patients [9]. Comparable results were obtained in similar studies from China and Japan [10,11]. Interestingly, when we analyzed the responses from anesthesiologists using a survey similar to that used in the above-mentioned studies, we detected that their perception of preoperative evaluation was more favorable than that reported by their patients. (Table 4). Thus, 75% of anesthesiologists in our study reported having frequently or almost always advised patients about the health risks of smoking, whereas the data obtained from patients showed that only 31% actually did so. Furthermore, 75% reported having offered resources to help patients quit, whereas the data obtained from patients showed that no anesthesiologist had offered such support (Table 4). This overestimation of counseling activities reaches and even exceeds the results observed in the pilot study by Warner et al., which was completed after an intensive educational project to promote smoking cessation counseling in preoperative anesthesia evaluation [12].

These discrepancies are probably due to the complexity involved in implementing tobacco cessation interventions in clinical practice. They could also result from the methodology used, namely, self-reporting by anesthesiologists compared with a direct patient survey. Some authors have suggested that direct observation of clinical practice could be the gold standard for measuring counseling activities because physician reports typically overestimate counseling activities, as we have demonstrated in our study [15].

Jaen et al. used direct observation to evaluate patterns of tobacco cessation counseling in 91 cigarette smokers who visited 20 family physicians [14]. The authors identified five counseling patterns, ranging from appropriate to no provision of tobacco cessation counseling. Only 21% of patients were appropriately counseled, even though more than half of the physicians demonstrated that they had the skills needed to provide a high-quality brief cessation intervention [14]. A recent study examined the degree to which two different methods (motivational or not motivational) conducted by primary care physicians promoted smoking cessation [16]. The physician participants were blinded to the study purpose. The authors showed that 56% of smokers received motivational tobacco cessation counseling, which is a high rate compared with that in previous reports; however, the authors recognized that implementation of motivational training programs to smokers to improve physicians’ capacity to counsel patients, still remains necessary [16].

We do not know the reasons for the infrequent interventions of the anesthesiologists observed in our study. Similar to other specialties, we might suspect an excessive caseload, lack of expertise in counseling smokers, lack of available pharmaceutical therapies, lack of training or education on smoking cessation, and, probably, the perception that several months of abstinence are necessary for a full benefit to be reached, particularly regarding pulmonary complications. Remarkably, anesthesiologists prescribed β_2_ adrenergic aerosols and corticosteroids more than they recommended other measures in smokers. This finding probably reflects concern over perioperative respiratory events, such as bronchospasm associated with airway manipulation [17,18].

In 2010, a total of 22,383 patients underwent surgery at our hospital. Surgery included ambulatory procedures, endoscopy, invasive radiologic procedures and other practices. Almost all of these patients were seen in the preoperative clinic or visited by an anesthesiologist if the patient was hospitalized. Given that 18.6% of patients in the present report were current smokers, almost 4,200 patients would have been eligible for counseling at the preoperative anesthesia clinic. Experts in preoperative smoking cessation counseling recommend that when anesthesiologists see smokers before surgery, they should perform the minimal Ask–Advise–Refer intervention to reduce perioperative risk [9], which could be considered standard of care. This standard of care was not present in our study findings.

Patients hospitalized for surgery have higher tobacco abstinence rates after hospital discharge than the general population, particularly if they are undergoing major procedures [18-21]. In our study, 34% of smokers were scheduled for major surgery, which provided an excellent opportunity for counseling on postoperative smoking cessation. At our institution, the mean time available from preoperative visit to surgery is 30 days, which would allow a considerable period of abstinence from smoking before surgery. However, we provide no specific smoking policies adapted to surgical patients (smoking cessation materials and pharmaceutical treatment options) or to the follow-up of these patients. An approach including a systematic integrated smoking checklist into the preanesthetic files, completed with regular meetings dedicated to inform about the risk of smoking in the perioperative period, could improve the anesthesiologist management of the smoker patient’s underling surgery.

Our study had several limitations. While direct observation of clinical practice has the advantage of reducing recall bias and increasing objectivity, it is subject to certain limitations. First, the number of smokers evaluated in our study was small, but although lower than that recorded in national surveys (around 33.7%), it was sufficient to evaluate the practices of anesthesiologists in tobacco cessation counseling. A second limitation is that we do not know the state of the patients with regard to quitting smoking. If the anesthesiologist who evaluated the patient considered that the patient was in the pre-contemplative stage (not seriously considering quitting smoking), they may have determined that it would be unlikely for them to move to an action stage in the brief time before surgery. However, surgery is considered as a teachable moment that increases perception of personal risk, and represents an opportunity to emphasize to patients the importance of smoking cessation.

A third limitation is that the anesthesiologists represented here were not specifically selected and, therefore, cover several surgical specialties, which could lead to an underestimation of the frequency with which assistance with smoking cessation was offered. In fact, anesthesiologists working in thoracic surgery were the most actively involved in counseling smokers. Finally, we recognize that we cannot predict the generalizability of our results to other settings with a proactive program for providing perioperative smoking cessation counseling and an efficient follow-up process after surgery.

## Conclusions

This study shows significant discrepancies between direct patient surveys of preoperative smoking cessation counseling activities by anesthesiologists and the self-reported perceptions of the anesthesiologists. Future studies are urgently needed to evaluate the provision of educational materials and other interventions to improve smoking cessation counseling rates among anesthesiologists and to narrow these discrepancies. This study reveals that many opportunities to integrate smoking cessation counseling into preoperative evaluation are missed.

## Abbreviation

ASA: American Society of Anesthesiologists
